# Association between plasma ADAMTS13 levels/activity and the risk of ischemic stroke and outcomes of recanalization therapies: a systematic review and meta-analysis

**DOI:** 10.3389/fneur.2025.1687480

**Published:** 2026-07-20

**Authors:** Yanyan Li, Rui Ding, Peng Gao, Ziqi Liu, Xiaorui Pei, Lifeng Piao

**Affiliations:** 1 Department of Neurology Ward, Chaoyang Central Hospital, Chaoyang, Liaoning, China; 2 Department of Laboratory of Molecular Biology, Chaoyang Central Hospital, Chaoyang, Liaoning, China; 3 Department of Reproductive Medicine, Chaoyang Central Hospital, Chaoyang, Liaoning, China; 4 Class 243, Clinical Medicine Major, Xinhua Clinical College, Dalian University, Dalian, Liaoning, China; 5 Department of Respiratory Ward, Chaoyang Central Hospital, Chaoyang, Liaoning, China

**Keywords:** acute ischemic stroke, ADAMTS13, NIHSS score, intravenous thrombolysis (IVT), endovascular thrombectomy (EVT), futile recanalization, prognosis, biomarkers

## Abstract

**Background:**

Large vWF (von Willebrand Factor) multimers are associated with ischemic stroke, and itsthrombogenicity is controlled by ADAMTS13 (a Disintegrin and Metalloprotease with repetitions in the ThromboSpond in motif). ADAMTS13 can cleave large vWF multimers into smaller, less procoagulant forms, preventing spontaneous platelet-thrombus formation. Extensive research has been conducted on the connection between ischemic stroke and ADAMTS13 activity as well as antigen levels, but got inconsistent conclusions. More recently, contradictory findings have been reported regarding the relationship between ADAMTS13 antigen levels or activity and recanalization therapy such as intravenous thrombolysis and mechanical thrombectomy.

**Methods:**

A thorough literature search was conducted from July 2th, 2001 to April 7th, 2025, utilizing various online databases including PubMed, Cochrane Library, Web of Science, and EMBASE. This meta-analysis aimed to synthesize observational evidence on the association between ADAMTS13 antigen or activity and stroke, and to evaluate its potential influence on recanalization outcomes following intravenous thrombolysis or mechanical thrombectomy. The pooled relative risk (RR) of stroke and the standardized mean difference (SMD) of the ADAMTS13 for stroke versus control subjects, as well as their corresponding 95% confidence interval (CI) were calculated. Eighteen studies with a total of 8,993 participants were included into this meta-analysis.

**Results:**

(1) ADAMTS13 activity was lower in patients with ischemic stroke when compared with controls, the SMD was −1.12 (95%CI: −1.20, −1.03, *P* < 0.001). Patients with ischemic stroke had decreased ADAMTS13 plasma antigen levels when compared to controls, the SMD was −1.32 (95%CI: −1.49, −1.15, *P* < 0.001). (2) The multivariable-adjusted relative risk (RR) indicated that lower ADAMTS13 levels were associated with an elevated risk of ischemic stroke, the RR was 1.75 (95%CI: 1.4, 2.19, *P* < 0.001). (3) Ischemic stroke patients who achieved unfavorable outcomes following recanalization therapy exhibited lower baseline ADAMTS13 antigen levels compared to those with favorable outcomes, the SMD was −0.26 (95%CI: −0.51, −0.01, *P* = 0.04). No significant difference in baseline ADAMTS13 activity was observed between patients with favorable and unfavorable outcomes following recanalization therapy, the SMD was −0.05 (95%CI: −0.36, 0.26, *P* = 0.75). (4) The multivariable-adjusted relative risk (RR) indicated that lower baseline ADAMTS13 levels were significantly associated with unfavorable early neurological outcome following recanalization therapy in ischemic stroke. However, no significant association was observed between baseline ADAMTS13 levels and 90-day functional outcomes in ischemic stroke patients without recanalization therapies, with a risk ratio (RR) of 0.92 (95%CI: 0.75, 1.12, *P* = 0.009).

**Conclusion:**

ADAMTS13 activity and ADAMTS13 plasma antigen levels were lower in patients with ischemic stroke. Reduced ADAMTS13 levels were significantly correlated with an increased risk of ischemic stroke. Meanwhile, baseline ADAMTS13 antigen levels were lower in patients with unfavorable outcomes in ischemic stroke patients who undergoing recanalization therapy. Notably, lower ADAMTS13 levels are associated with unfavorable early neurological outcome after recanalization therapy in acute ischemic stroke rather than 90-day prognosis.

**Systematic review registration:**

https://www.crd.york.ac.uk/PROSPERO/view/CRD420251229650, Identifier: CRD420251229650.

## Highlights

- Reduced antigen levels and activity of ADAMTS13 were significantly linked to an increased risk of ischemic stroke.
- Meanwhile, lower baseline ADAMTS13 antigen levels were associated with unfavorable outcomes in ischemic stroke patients who undergoing recanalization therapy, especially with unfavorable early neurological outcome but not with 90-day prognosis.

## Introduction

Ischemic stroke is a one of the leading cause of mortality, disability and death. While many risk factors have already been identified, the potential pathogenesis of ischemic stroke remains unclear and treatment options are limited. Therefore, identifying new risk factors for stroke and exploring novel therapeutic approaches are of great importance. ADAMTS13 is a metalloprotease that cleaves ultra-large Von Willebrand Factor (ULVWF) into smaller, less active ones, less likely to cause blood clotting forms, which is linked to several diseases (1). The *in vivo* synthesis and activity of ADAMTS13 are subject to a sophisticated regulatory system. Primarily produced by hepatic stellate cells and endothelial cells, the protease circulates at a stable baseline level. Its activity is not constitutive but is dynamically regulated (2, 3). The primary trigger for its function is a conformational change in its substrate, von Willebrand factor (VWF), which occurs under elevated shear stress, exposing the cryptic cleavage site. Beyond this substrate-driven activation, ADAMTS13 levels can be influenced by inflammatory signals, for example, interleukin-6 (IL-6) is known to enhance its synthesis. On the other hand, its activity is critically impaired by inhibitory autoantibodies, which are the hallmark of acquired TTP (4). Other factors, including proteolytic cleavage and oxidative stress, can also inactivate the enzyme. A disruption in this delicate equilibrium between synthesis, activation, and inhibition is increasingly recognized as a key factor in the thrombotic cascade of ischemic stroke.

In recent years, ADAMTS13 has attracted increasing attention in numerous diseases. ADAMTS13 assessment includes two key parameters: ADAMTS13 antigen levels (refers to the concentration ofADAMTS13 protein in the blood, reflecting the quantitative presence of the enzyme) and ADAMTS13 activity (which measures the catalytic activity of ADAMTS13, specifically its ability to cleave von Willebrand factor (vWF)). The cleavage of VWF has been discovered to reduce both thrombosis and inflammation (5–7). A complete diagnostic workup for ADAMTS13 deficiency in ischemic stroke patients should include both activity and antigen testing. While the activity level identifies the functional deficit, the antigen level is necessary for a differential diagnosis. This combined approach differentiates a quantitative deficiency (parallel reductions in both measures, implying under production or consumption) from a qualitative deficiency (disproportionately low activity with normal antigen levels, pointing to inhibitors or dysfunctional variants). This distinction has direct clinical utility: it clarifies the thrombotic mechanism, may guide targeted interventions (like immunosuppression), and refines prognostic outlook. Consequently, integrating both parameters is fundamental for precise diagnosis and informed patient management (8). Numerous clinical and experimental studies have investigated the association between ADAMTS13 deficiency and arterial thrombotic events, particularly ischemic stroke and acute myocardial infarction (9). Some case–control studies suggested that ADAMTS13 levels and activity are decreased in patients with ischemic stroke, however, other studies had yielded conflicting results (10–12). Meanwhile, many studies have reported conflicting evidence regarding the association between ADAMTS13 levels and patient prognosis, stroke severity, or its potential role as an ischemic stroke risk factor (10, 13–16).

Intravenous thrombolysis with rt-PA and mechanical thrombectomy following stroke have become the main treatment choices for acute ischemic stroke, which closely correlated with the prognosis of ischemic stroke (17, 18). However, intravenous thrombolysis with rt-PA and mechanical thrombectomy can have varied clinical results and increase the risk of cerebral hemorrhage, therefore the discovery of biomarkers for predicting responsiveness to reperfusion efforts is critical. Some studies have demonstrated significant variations in both baseline ADAMTS13 antigen levels and activity among ischemic stroke patients undergoing intravenous thrombolysis and mechanical thrombectomy, which were associated with differential clinical outcomes (19–21). It is uncertain whether ADAMTS13 antigen levels and activity might predict response to in individuals who undergo intravenous thrombolysis and mechanical thrombectomy.

Therefore, our present meta-analysis represents the first comprehensive examination of ADAMTS13 antigen levels and activity in relation to ischemic stroke. Our study specifically investigates: (1) the potential role of ADAMTS13 as a risk factor for ischemic stroke, and (2) the prognostic value of ADAMTS13 levels and activity in patients who received recanalization therapies.

## Materials and methods

### Search strategy and study selection

**Figure 1:**
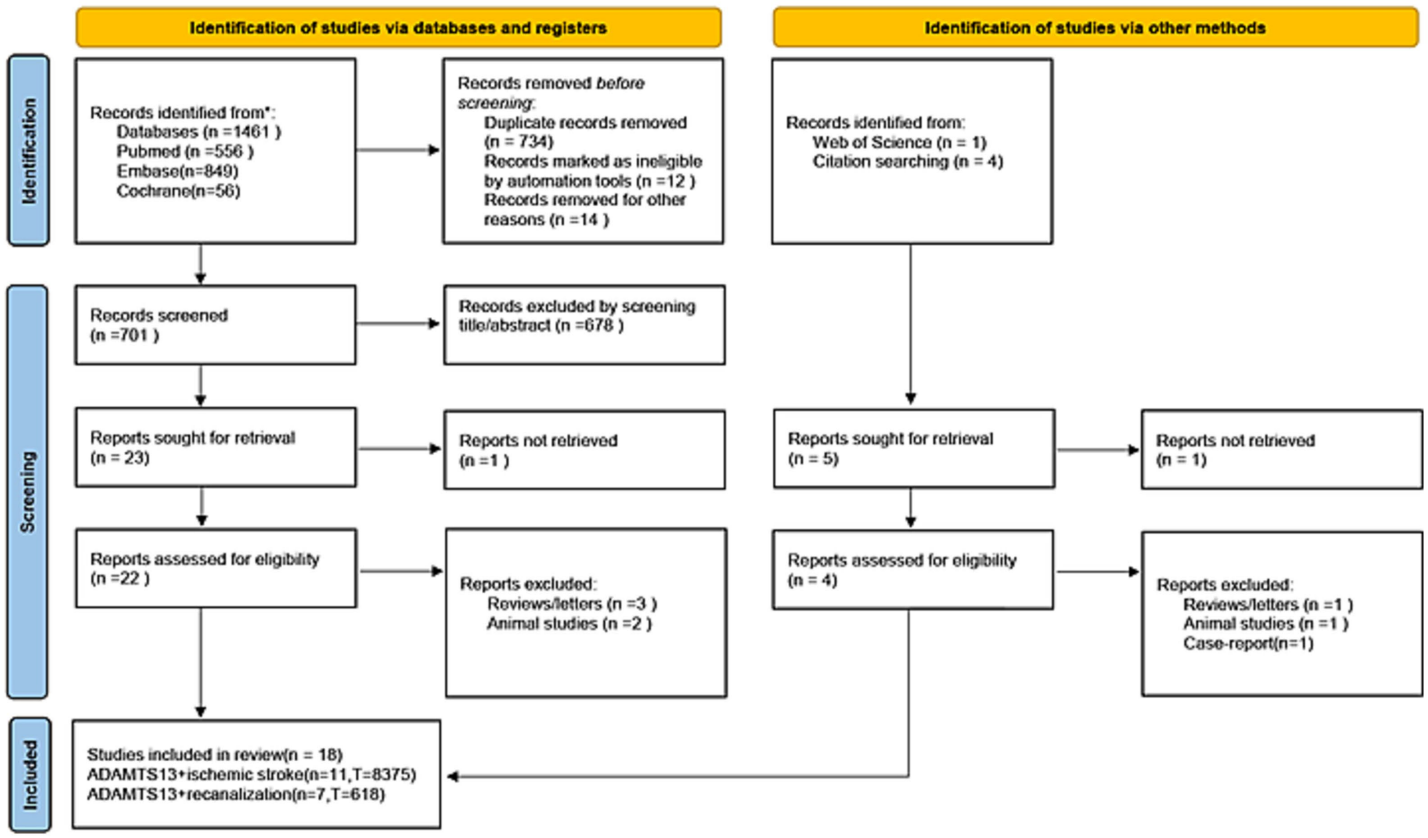
Preferred Reporting Item for Systematic Reviews and Meta-Analysis (PRISMA) guideline.

A comprehensive literature search was conducted in the PubMed, Cochrane Library, Web of Science, and EMBASE databases from July 2, 2001, to April 7, 2025. Additional data were acquired from Google Scholars reference list of included studies and possibly related papers, which were not detected during the literature search. The following search phrases and keywords were connected by “and” or “or” in the search strategy: ischemic stroke/cerebral infarction/cerebrovascular disease /ADAMTS13/ a Disintegrin and Metalloprotease with ThromboSpondin motif repeats 13. Two writers independently chose publications from the resultant list, screening the article titles and abstracts. The articles that satisfied the screening requirements were extensively assessed in full text according to the eligibility criteria. The study followed the PRISMA standard, as seen in Figure 1. The study was preregistered in PROSPERO (CRD420251229650).

### Data extraction and study quality

#### Inclusion criteria

The search approach focused on public data and English language. Publications were included when: First, prospective cohort studies or case–control studies; Second, the study concerning the relationship between ADAMTS13 and stroke; Third, the inclusion criteria for this systematic review were strictly defined according to the PICOS framework, as detailed below.

*P(Population):* Adult patients (≥18 years) with acute ischemic stroke, confirmed by neuroimaging, and/or who underwent revascularization therapy(intravenous thrombolysis and mechanical thrombectomy). *I(Intervention/Exposure)*: Measurement of ADAMTS13 activity or levels within 24 h of stroke onset. The exposed group was defined as patients with low ADAMTS13 levels or in the lowest quantile. *C(Comparison):* Healthy controls(In our study, the control group was defined as healthy individuals with no history of stroke or other major neurological, cardiovascular, or systemic diseases). *O(Outcomes):* The primary outcomes were mTICI≥2b post-thrombectomy or functional dependence (mRS ≤ 2 at 90 days)or neurologic improvement at 24 h (defined as decrease in the NIHSS score by ≥4 points). *S(Study design)*: Observational cohort studies (both prospective and retrospective) and relevant arms of randomized controlled trials.

#### Exclusion criteria

We excluded: (1) duplicate or irrelevant papers; (2) reviews, letters, case reports, and comments; (3) non-original research; (4) studies involving non-human subjects; (5) unpublished or non-peer-reviewed studies.

#### Outcome definitions

The post-intervention reperfusion grade was assessed using the modified Treatment in Cerebral Infarction (mTICI) score. The clinical outcome was evaluated using the modified Rankin scale (mRS) and NIHSS score (NIH Stroke Scale). Classification of patients was performed according to post-recanalization mTICI, mRS, and NIHSS scores: *(1) Favorable outcomes*: mTICI ≥2b post-thrombectomy or functional dependence (mRS ≤ 2 at 90 days) or neurologic improvement at 24 h (defined as decrease in the NIHSS score by ≥4 points); *(2) Unfavorable outcomes:* mTICI <2b post-thrombectomy or functional dependence (mRS ≥ 3 at 90 days).

The prognosis can be categorized into: (1) early outcome (based on NIHSS score or early post-reperfusion blood flow recovery): neurological improvement (defined as a reduction of ≥4 points on the NIHSS or mTICI ≥2b assessed at 7 days; (2) 90-day prognosis (based on mRS score) post-thrombectomy) assessed at 24 h, or functional independence (defined as a mRS score of 0–2).

#### Data extraction

Two reviewers independently gathered data from eligible studies, including authors, year of publication, research design, sample size, biomarker levels, patient outcomes, and Newcastle-Ottawa Scale (NOS).

#### Quality assessment

Each author evaluated the quality and risk of bias in the studies by using the Newcastle-Ottawa Scale (NOS) (22). Any inconsistencies were addressed through discussion. The scoring method is divided into three domains: sample cohort selection, cohort comparability, and result evaluation. Studies were categorized as poor or high quality based on ratings ranging from 0–4 and 5–9 points.

## Statistical analysis

The meta-analysis was carried out using Review Manager software 5.3 and STATA version 17.0. For continuous outcomes, the pooled treatment effects (e.g., standardized mean difference) are presented with their 95% confidence intervals (CIs). Descriptive data for continuous variables within individual studies are summarized as mean ± standard deviation. We estimated the mean and standard deviation using the Wan et al. approach if the data in the included studies were given as median and quartile values. The application of the Wan et al. method was governed by strict prerequisites (23). Its core requirement was that it was exclusively applied to studies that reported medians with interquartile ranges or ranges, but did not report means and standard deviations. For all studies that provided means and standard deviations directly, the original data were utilized without any conversion. I^2^ statistics were utilized to evaluate data In review heterogeneity. When I^2^ exceeded 50%, the random-effects model was employed to assess the results of the included research. When I^2^ less than 50%, a fixed-effect model was used instead. Forest plots were used to depict odds ratios, and summary estimates were calculated using random effects models to account for anticipated clinical and methodological heterogeneity among the included studies. Significant *p*-values were those below 0.05, and the analysis also gave 95% confidence intervals. The overall odds ratio was calculated using a mixed logistic regression model incorporating random treatment effects. Forest plots were utilized to calculate the pooled effect sizes and 95% confidence intervals. The publication bias was measured using Eggers test and a funnel plot, and its symmetry was examined visually.

## Results

### Literature search and study characteristics

#### Identification of studies via databases and registers

A total of 1,461 records were identified through database searching. After removing 734 duplicate records, 727 records were screened. Automation tools excluded 12 records, and a further 14 records were removed for other reasons, leaving 701 records for title and abstract screening. Of these, 678 records were excluded, resulting in 22 reports that were sought for retrieval and assessed for eligibility. After excluding 3 reviews/letters and 2 animal studies, 1 report was not retrieved. Subsequently, 17 studies were eligible for inclusion.

#### Identification of studies via other methods

Five additional records were identified through other sources. After excluding 1 review/letter, 1 animal study, and 1 case report, 2 reports were sought for retrieval. One of these could not be retrieved, leaving 1 study eligible for inclusion. In total, 18 studies were included in the systematic review. Table 1 lists the features of the included studies, Figure 1 shows the literature search flow chart. Consequently, 11 studies involving ADAMTS13 and ischemic stroke totaling 8,375 individuals, 7 studies with 618 participants involving ADAMTS13 and recanalization therapy were submitted for meta-analysis and qualitative examination (Table 1). Quality rating varied from six to nine. All featured records were rated as moderate to high quality.

**Table 1:** Characteristics of the included studies.

Study and year	Country	*N* total	ADAMTS13 assay	Cases	Controls	OR	Quality (NOS)
ADAMTS13 and stroke							
Qu 2015 (28)	China	197	Activity (U/mL)	108.65 ± 10.49	117.83 ± 8.57	2.4 (1.06–5.44)	8
Taylor 2020 (29)	United Kingdom	292	Activity	84.68 ± 15.59	94.76 ± 19.49	NA	6
Kawano 2016 (11)	Japan	89	Activity	87.66 ± 9.42	91.22 ± 5.35	NA	8
Tamara 2006 (16)	The Netherlands	249	Activity	0.96 ± 0.41	1.03 ± 0.44	1.7 (0.7–3.9)	7
McCabe 2015 (14)	UK	75	Activity	73.32 ± 26.48	92.25 ± 27.23	NA	9
Andersson 2012 (7)	The Netherlands	793	Antigen level (ug/mL)	1.02 ± 0.30	1.12 ± 0.39	3.1 (1.6–5.8)	6
Bongers 2009 (15)	The Netherlands	441	Activity	95.85 ± 8.28	109.45 ± 5.32	1.76 [0.90–3.44]	8
			Antigen level	78.66 ± 9.15	97.35 ± 5.07	NA	
Denormel 2017 (13)	Germany	189	Antigen level	82.6 ± 21.0	99.6 ± 24.5	NA	7
Makris 2025 (30)	Greece	29	Activity	72.07 + −21.85	68.98 + −16.26	NA	7
Sonneveld 2015 (12)	The Netherlands	5,941	Activity	70.3 ± 0.17	91.9 ± 17.8	1.52 (1.15–2.02)	9
Dong 2008 (9)	China	80	activity	65.4 ± 15.8	81.7 ± 13.9	NA	7
			Antigen level (U/L)	702 ± 155	878 ± 198		
ADAMTS13 and recanalization				Recanalization	Futile-recanalization		
Putzer 2019 (20)	Germany	43	IVT activity			1.298 (1.00–1.68)	7
Su 2020 (43)	China	163	IVT antigen level (ng/mL)	1578.3 ± 395.4	1458.4 ± 323.3	0.92 (0.75–1.13)	8
Lu 2022 (31)	China	74	IVT antigen level (pg/mL)	809.88 ± 187.51	564.69 ± 82.21	0.07 (0.005–0.991)	9
Bustamante 2018 (21)	Spain	72	IVT activity	76.01 ± 5.36	69.98 ± 4.79	6.76 (1.52–30.02)	8
		59	EVT antigen level	1340.5 ± 128.2	1590.5 ± 121.8	67.4 (1.4–3282.1)	
Zang 2020 (44)	China	61	EVT activity	–	–	0.04 (0.002–0.54)	7
Prochazka 2017 (42)	USA	105	EVT activity	63.87 ± 17.45	75.14 ± 17.71	NA	8
Schuppner 2017 (19)	Germany	41	EVT activity	–	–	0.009	8

### The association between ADAMTS13 levels/activity and ischemic stroke

**Figure 2:**
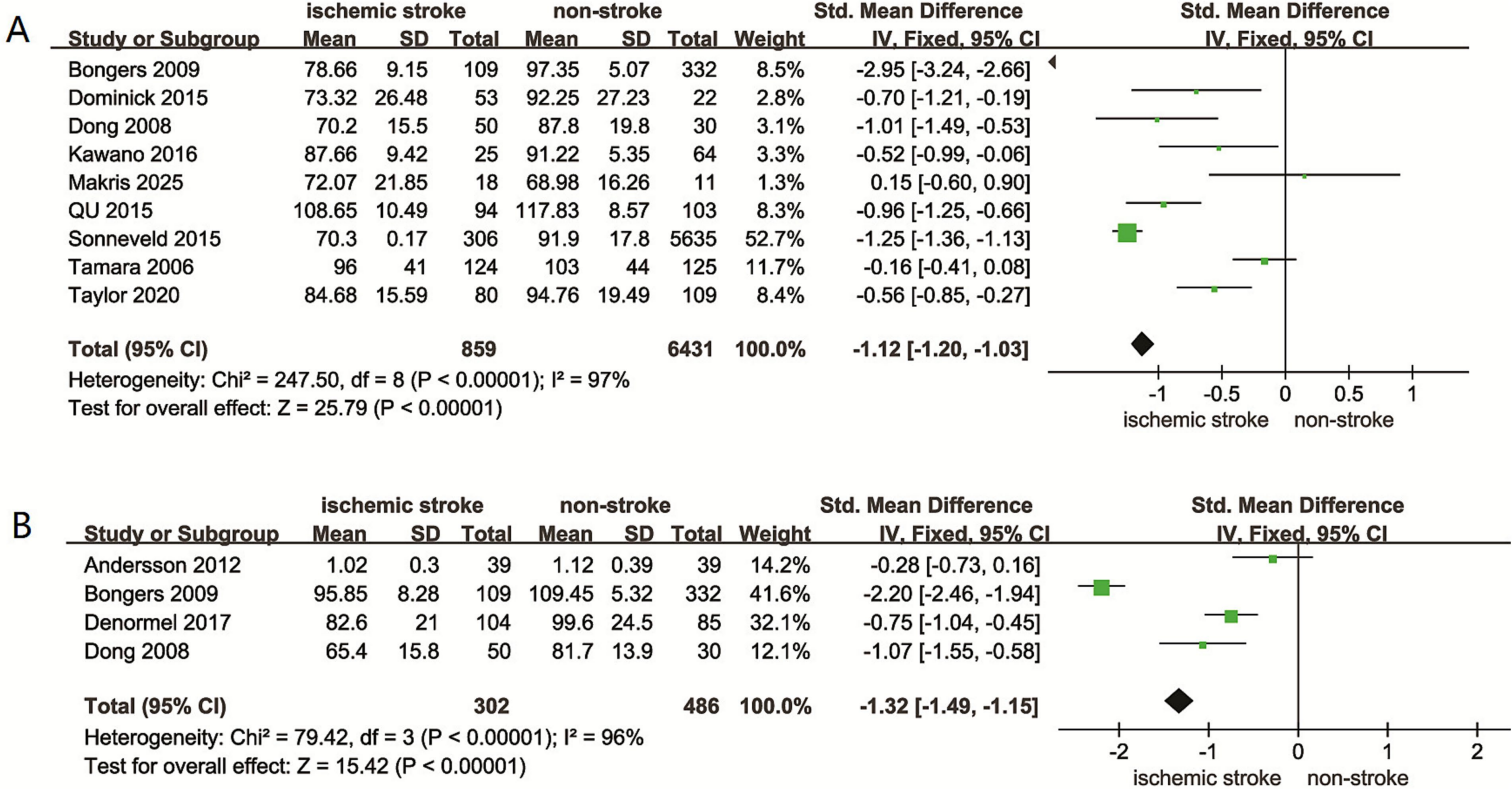
ADAMTS13 antigen levels and activity between ischemic stroke patients and controls. **(A)** ADAMTS13 activity between ischemic stroke patients and controls. **(B)** ADAMTS13 antigen levels between ischemic stroke patients and controls.

The present meta-analysis revealed that ADAMTS13 activity was lower in patients with ischemic stroke when compared with controls, the SMD was 1.12 (95%CI: −1.20, −1.03, *p* < 0.001) (Figure 2A). Patients with ischemic stroke had decreased ADAMTS13 plasma antigen levels when compared to controls, the SMD was −1.32 (95%CI: −1.49, −1.15, *p* < 0.001) (Figure 2B).

**Figure 3:**
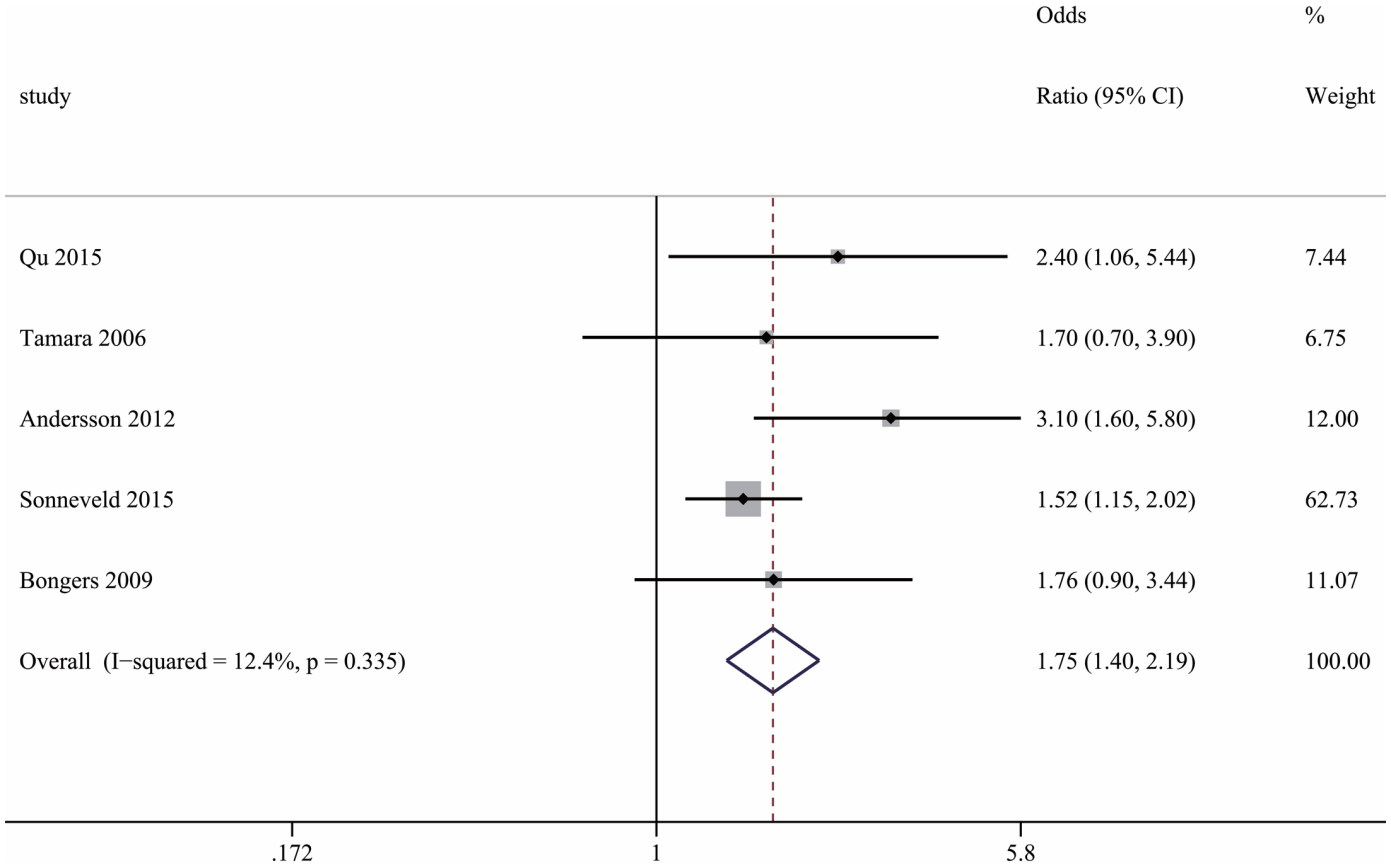
Association between ADAMTS13 levels and ischemic stroke.

The multivariable-adjusted relative risk (RR) indicated that decreased levels of ADAMTS13 are correlated with an increased risk for ischemic stroke (RR = 1.75 (95%CI: 1.4, 2.19), *p* = <0.001) (Figure 3).

### The association between ADAMTS13 levels/activity and ischemic stroke prognosis in patients who received recanalization therapy

**Figure 4:**
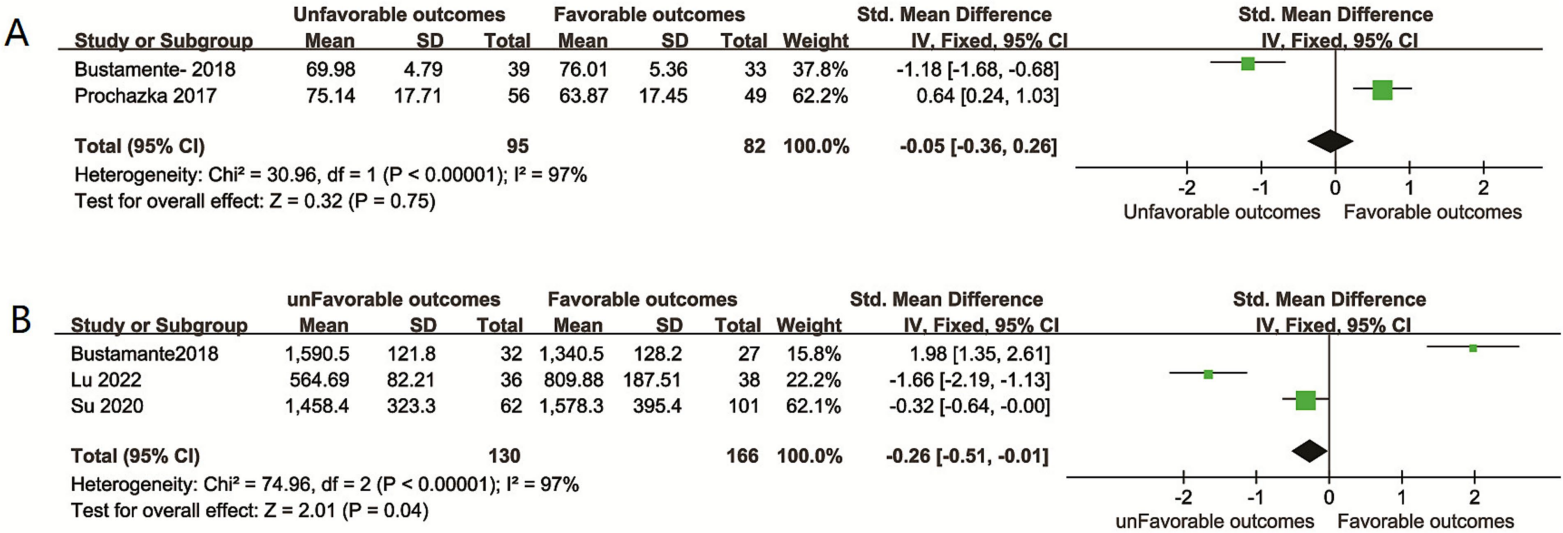
ADAMTS13 levels and activity between favorable outcomes and unfavorable outcomes in patients who received recanalization therapy. **(A)** ADAMTS13 activity between favorable outcomes and unfavorable outcomes. **(B)** ADAMTS13 antigen levels between favorable outcomes and unfavorable outcomes.

There was no significant difference in baseline ADAMTS13 activity between patients with favorable outcomes and unfavorable outcomes receiving recanalization therapy, the SMD was −0.05 (95%CI, −0.36, 0.26, *p* = 0.75) (Figure 4A). Patients who achieved unfavorable outcomes in ischemic stroke patients undergoing recanalization therapy had lower baselineADAMTS13 antigen level, the SMD was −0.26 (95%CI: −0.51, −0.01, *p* = 0.04) (Figure 4B).

**Figure 5:**
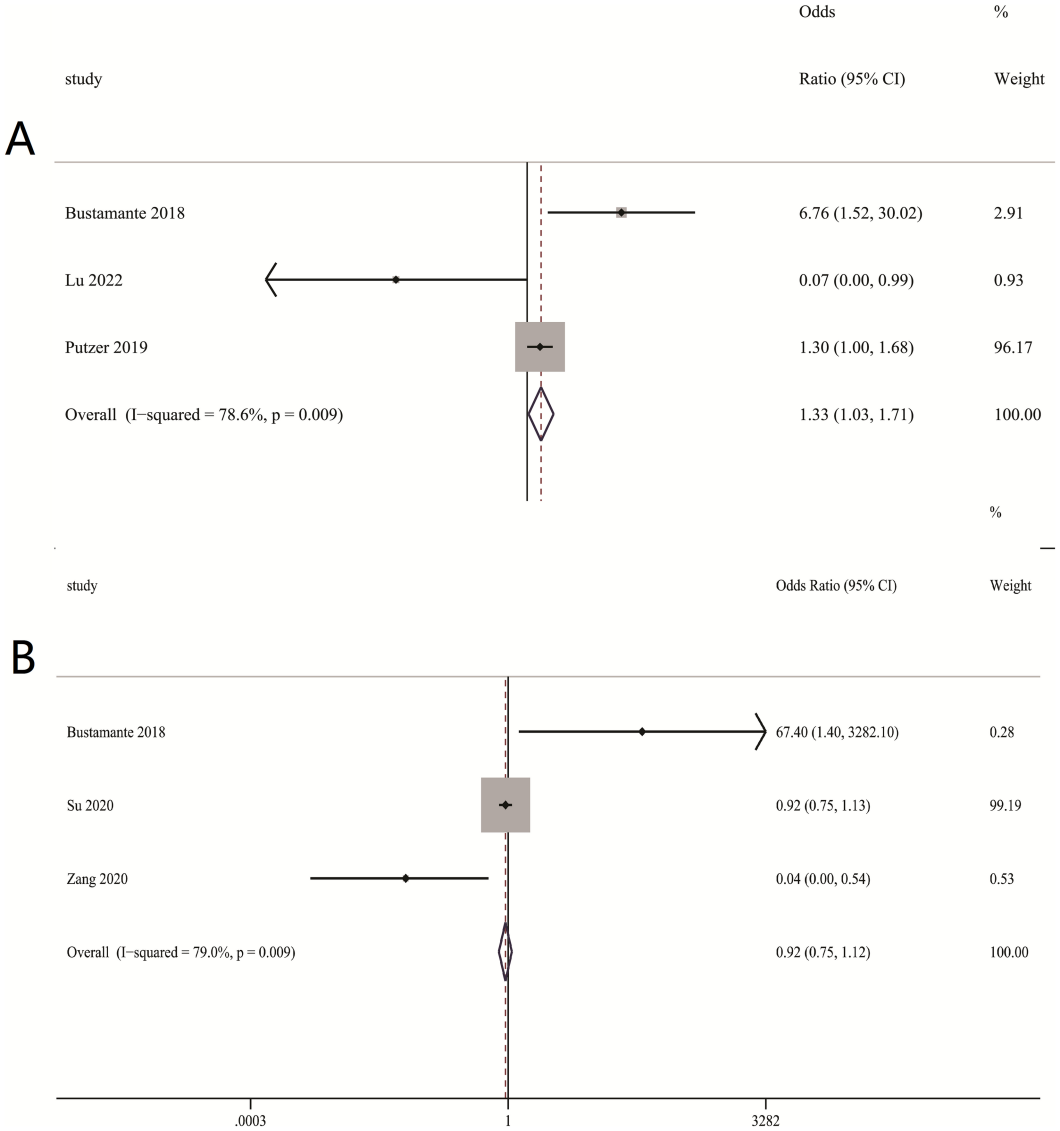
The connection between ADAMTS13 levels and ischemic stroke prognosis. **(A)** ADAMTS13 levels and early prognosis. **(B)** ADAMTS13 levels and 90-days prognosis.

The multivariable-adjusted relative risk (RR) indicated that lower baseline ADAMTS13 levels were associated with unfavorable early neurological outcome after recanalization therapy in ischemic stroke patients, with RR of 1.33 (95%CI: 1.03, 1.71, *p* = 0.009) (Figure 5A); However, there was no association between baseline ADAMTS13 levels and clinical outcomes of ischemic stroke patients at 90 days, with RR of 0.92 (95%CI: 0.75, 1.12, *p* = 0.009) (Figure 5B).

### Publication bias

**Figure 6:**
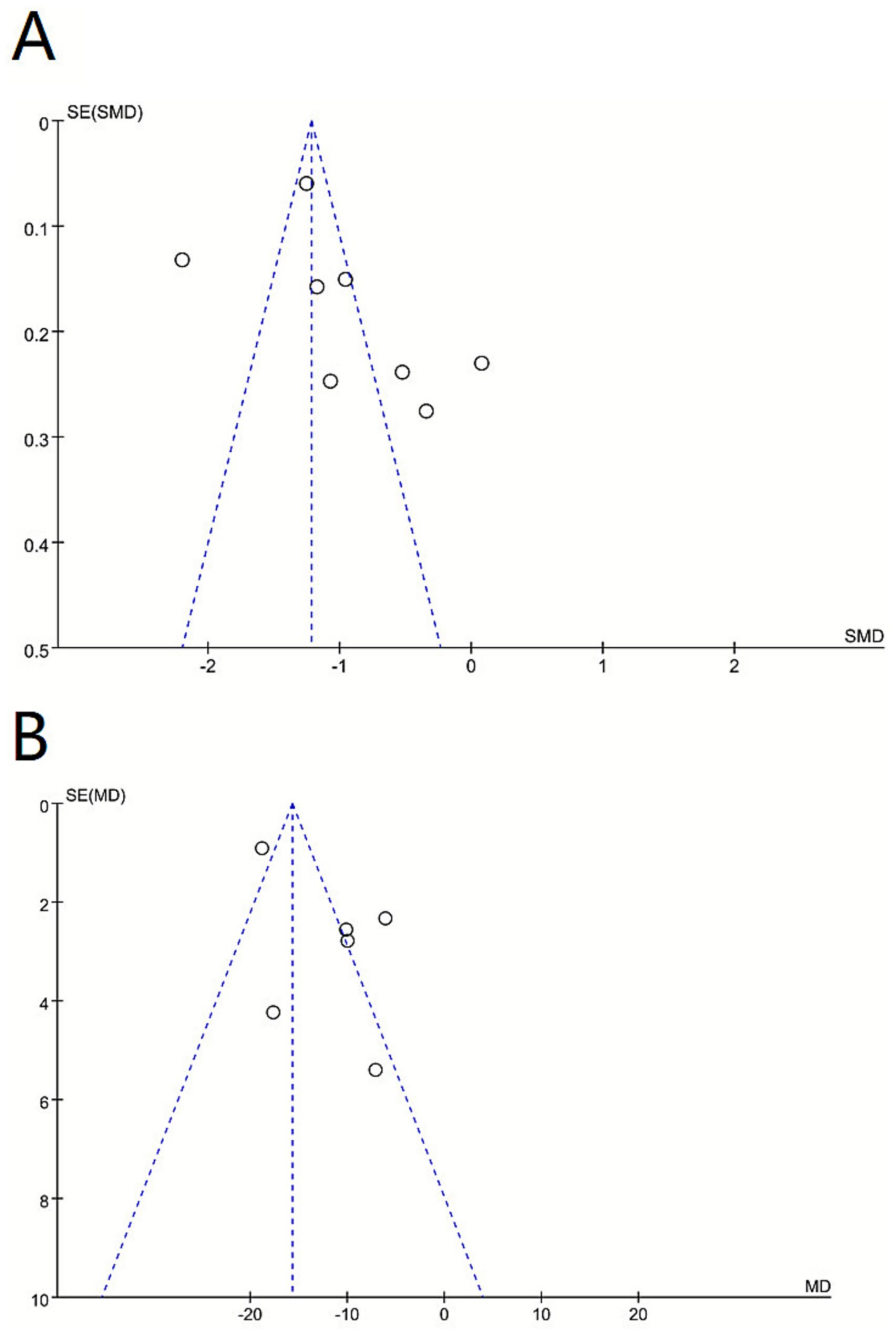
Funnel-plot analysis. **(A)** Publication bias in studies on ADAMTS13 levels and ischemic stroke. **(B)** Publication bias in studies on ADAMTS13 levels and recanalization.

*ADAMTS13 and Acute Cerebral Infarction* (Figure 6A): The funnel plot was asymmetrical, suggesting potential bias; however, Egger’s test did not indicate significant evidence (*t* = 1.23, df = 8, *p* = 0.254).

*ADAMTS13 and Recanalization Therapy* (Figure 6B): The funnel plot was symmetrical, and Egger’s test confirmed no significant publication bias (*t* = −0.14, df = 3, *p* = 0.898).

## Discussion

Our present meta-analysis indicates that ADAMTS13 antigen levels and activity are lower in ischemic stroke patients than in controls. There is a negative correlation between ADAMTS13 levels and ischemic stroke, indicating that ADAMTS13 may play a role in the development of ischemic stroke. Likewise, the present meta-analysis demonstrates that patients who achieved unfavorable outcomes in ischemic stroke patients who undergoing recanalization therapy had lower baseline ADAMTS13 antigen level rather than lower ADAMTS13 activity. Similarly, lower ADAMTS13 levels are associated with unfavorable early neurological outcome in patients receiving recanalization therapy, but not with 90-day prognosis.

Lower levels of ADAMTS13 can result in the localized buildup of ultra-large VWF in areas of vascular damage, favoring the development of thromboembolic occlusions (1, 2). These findings revealed a possible association between different types of arterial blood clotting and ADAMTS13 levels. Some recent studies discovered a connection between a regional disparity in VWF and ADAMTS13 levels and coronary stenosis patients with ST-elevation myocardial infarction (24–26). Reduced ADAMTS13 antigen levels were associated with increased risk of myocardial infarction (27). Notably, some studies focus on the relationship between ADAMTS13 levels and ischemic stroke, but got inconsistent conclusions (5–12, 14–16). Multiple studies have found an association between lower ADAMTS13 activity and ischemic stroke (14, 28). Furthermore, this activity has been shown to be significantly reduced in ischemic stroke patients versus controls (29). In the Rotterdam study, a population-based cohort study with 6,130 participants, ADAMTS13 activity was lower in ischemic stroke patients, which showed reduced ADAMTS13 activity predisposes to stroke (12). It has been demonstrated that reduced ADAMTS13 levels are associated with ischemic stroke (11). Evidence from previous studies confirms a clear association between lower ADAMTS13 activity and acute ischemic stroke (8, 9). Contrasting results have been reported regarding ADAMTS13 levels in ischemic stroke, with one study finding slightly but not significantly lower levels, and another reporting higher activity compared to controls (16, 30). Notably, some studies had also explored the association between ADAMTS13 antigen levels and ischemic stroke. A study examined the associations of both ADAMTS13 antigen levels and ADAMTS13 activity with acute ischemic stroke, revealing that ADAMTS13 activity and ADAMTS13 antigen levels were significantly lower in ischemic stroke patients than the controls (15). Similar results were observed in another study, further supporting the link between ADAMTS13 antigen levels and stroke (13). In agreement with previous findings, our meta-analysis demonstrated significantly lower levels of both ADAMTS13 activity and antigen in the ischemic stroke population compared to controls (15). Meanwhile, our systematic review confirmed that there was a negative association between ADAMTS13 and ischemic stroke, indicating that ADAMTS13 may play a role in the development of ischemic stroke. Reduced ADAMTS13 activity and antigen levels not only predispose to ischemic stroke but may also aggravate its pathogenesis and prognosis, highlighting the need for additional Research (31). Both the antigen level and enzymatic activity of ADAMTS13 are subject to influence by a range of pathological conditions and comorbidities. For example, a population-based study demonstrated that patients with liver disease or renal insufficiency show reduced ADAMTS13 antigen levels, attributable to impaired hepatic synthesis and compromised renal metabolic function, respectively (16). Thus, in future clinical studies, it is critical to consider patients underlying disease and current comorbidities that may affect ADAMTS13 expression or function, in order to reduce potential confounding.

The pathophysiological mechanisms underlying the association between reduced ADAMTS13 levels and ischemic stroke remain incompletely understood. Experimental studies have found that ADAMTS13 has a preventive function in the pathogenesis of atherosclerosis (32). For example, mice with ADAMTS13 gene depletion (−/−) exhibited greater infarctions than wild-type mice (6). However, infusion of ADAMTS13 into wild-type mice with ischemic stroke reduced infarct size by 30% and improved functional outcome (33). We hypothesized that ADAMTS13 plays a role in arterial thrombosis because reduced ADAMTS13 activity will result in less degradation of UL-vWF multimers activity. The decreased ADAMTS13 levels observed in acute ischemic stroke patients may be explained by the findings from previous studies (34, 35). We hypothesize that this phenomenon could result from the following pathophysiological mechanisms: (1) the impact of ADAMTS13 levels on the development and advancement of the atherosclerotic plaque (36); (2) reduced ADAMTS13 activity can result in reduced cleavage of big multimers of vWF (18); and (3) ADAMTS13 deficiency exacerbates thrombus amplification and deleterious post-thrombotic inflammatory pathways (37).

Current evidence supports the clinical benefits of recanalization therapy in acute cerebral infarction (38, 39), however, there are still individuals with poor neurological outcomes as a result of reperfusion failure, rehtrombosis, or secondary bleeding. Accordingly, several studies had explored potential biomarkers that may predict responsiveness to recanalization therapies in patients (40–42). Emerging evidence indicated that baseline ADAMTS1313 levels may influence the efficacy of recanalization therapies (intravenous thrombolysis and mechanical thrombectomy) in acute ischemic stroke patients, but yielded divergent conclusions. Some researcher conducted a study to analyze ADAMTS13 activity in relation to arterial recanalization therapy including intravenous thrombolysis and mechanical thrombectomy, which revealed that reduced ADAMTS13 activity was associated with poor response to recanalization therapies (21). An association has been observed between lower ADAMTS13 activity and poor early neurological outcome following intravenous thrombolysis (IVT) in ischemic stroke patients (20). However, there was no association of ADAMTS13 activity and 90-days functional outcomes in the aforementioned studies. Evidence on the association between baseline ADAMTS13 levels and clinical outcomes in acute ischemic stroke remains divergent. One study found no association between ADAMTS13 antigen levels and 90-day outcomes in patients receiving intravenous thrombolysis (IVT) (39). In contrast, other research has demonstrated that low ADAMTS13 activity is independently associated with unfavorable outcomes in patients undergoing endovascular treatment (19). Furthermore, supporting this association, lower ADAMTS13 antigen levels measured 72 h post-IVT have been significantly linked to poor prognosis and functional outcomes in IVT-treated patients (43). In another study, lower ADAMTS13 activity was independently associated with both 90-days prognosis and overall early complications significantly. Our systematic review categorized patient prognoses into favorable and unfavorable outcomes based on post intervention reperfusion grade and clinical outcome. Based on the assessment time points, prognosis was categorized into early prognosis (within 1 week) and 90-day prognosis. Our meta-analysis confirms that a lower baseline ADAMTS13 antigen level, but not activity, was specifically associated with unfavorable outcomes in ischemic stroke patients following recanalization therapy (44). Interestingly, multivariable-adjusted relative risk (RR) indicated a more specific role for lower ADAMTS13 levels in predicting unfavorable early neurological outcome as opposed to the 90-day prognosis following recanalization therapy, a finding supported by existing literature (17).

The pathophysiological processes underlying the relationship between low ADAMTS13 antigen levels or activity and unfavorable outcomes are not well understood. In animal investigations, reduced ADAMTS13 levels led to higher cerebral infarct volume after ischemic stroke. Notably, a comparison between ADAMTS13−/− and wild-type mice revealed that the knockout models exhibited significantly reduced blood flow in the ischemic cortical area following reperfusion (45, 46). This critical difference underscores the profound impact of complete ADAMTS13 deficiency on microvascular reperfusion injury, which may not be fully recapitulated in models of partial ADAMTS13 reduction. Clinical and experimental data demonstrate that ADAMTS13 deficiency exacerbates ischemic stroke by permitting unchecked VWF-mediated platelet aggregation at sites of endothelial injury (47). Therapeutically, restoring ADAMTS13 activity may improve recanalization and mitigate secondary microthrombosis—a dual mechanism supporting its translational potential. The observed disparity between knockout and wild-type models carries significant methodological implications. It suggests that the choice of animal model (e.g., complete knockout versus partial deficiency) can critically influence the observed pathophysiology, particularly concerning post-reperfusion microvascular integrity. Future studies should, therefore, be designed to directly compare these models in a standardized setting to dissect the specific roles of ADAMTS13 in macro-recanalization versus microvascular protection. Furthermore, investigating the efficacy of ADAMTS13 supplementation across these different models will be essential to fully understand its therapeutic potential and to inform the design of future clinical trials. In the present study, ADAMTS13 activity showed no significant association with long-term clinical outcomes. This observation is biologically reasonable, since ADAMTS13 constitutes merely one factor affecting recanalization therapy outcomes, which in turn explains only part of the variability in long-term functional improvement. Previous studies have documented that ADAMTS13 activity may normalize during the chronic phase (≥3 months) following ischemic stroke (14). A key finding emerging from our analysis is the differential prognostic utility of ADAMTS13 antigen and activity measurements over time. So we performed a more detailed analysis which revealed that baseline activity was a superior predictor of early clinical severity, whereas baseline antigen levels showed a stronger correlation with 90-day functional recovery. This temporal divergence has direct clinical relevance. The initial activity level likely captures the Intensity of the acute pro-thrombotic state, influencing infarct size and early neurological deficit. The antigen level, representing the total available protein, may be a surrogate for the body’s capacity to restore hemostatic. Balance and support endothelial integrity during the prolonged recovery phase. This distinction underscores that the two biomarkers are not interchangeable but are complementary, offering a more nuanced prognostic picture (48, 49). It suggests that therapeutic strategies aimed at modulating ADAMTS13 might need to consider both the immediate functional deficit and the underlying quantitative pool to optimize both short- and long-term patient outcome.

Our present meta-analysis has several important limitations. First, the majority of included studies employed case–control designs with inadequate matching of baseline characteristics or adjustment for potential confounding variables. Second, the assessment of ADAMTS13 lacked standardization, with measurements performed using either in-house assays or heterogeneous commercial kits, potentially introducing inter-assay variability. Third, the predominance of retrospective case–control studies in the current literature highlights the need for prospective cohort studies and randomized controlled trials to definitively establish the causal relationship between ADAMTS13 and ischemic stroke pathogenesis. Fourth, the absence of individual patient-level data precluded more sophisticated multivariable regression analyses to control for confounding factors. Finally, regarding data processing, the conversion methods we applied are based on data that potentially follow an on-normal distribution. Therefore, we treated the meta-analysis using the converted data as a conservative strategy aimed at maximizing data utilization, rather than assuming the converted data perfectly adhered to a normal distribution. Meanwhile, the inclusion of studies that required data transformation using the Wan et al. method is a potential limitation. While this approach maximizes data utilization, the accuracy of the converted estimates is contingent upon the underlying data distribution conforming to normality. This may be particularly problematic in studies with small sample sizes (e.g., N < 50), where the distribution of data is less stable and estimates of medians and ranges are less precise. Consequently, the incorporation of these converted values may have introduced additional heterogeneity and affected the precision of our pooled estimates. We hope future studies will pay more attention to the methodological consistency in data conversion. Therefore, well-designed randomized controlled trials(RCTs) that investigate the role of ADAMTS13 in stroke pathophysiology and its impact on recanalization therapy outcomes are urgently needed. Finally, our systematic review can not fully disentangle the influence of key patient characteristics—such as age, gender, comorbidities (like hypertension and diabetes), and the critical role of *in vivo* shear stress—from the observed associations between ADAMTS13 and stroke outcomes. These factors are established modulators of ADAMTS13 biology and may act as unmeasured confounders. It is also important to note a potential limitation. The results of the publication bias assessment for the association between ADAMTS13 and acute cerebral infarction presented a seemingly discordant picture: an asymmetrical funnel plot suggestive of potential bias, alongside a non-significant Egger’s test. This apparent discrepancy warrants careful interpretation. Firstly, the statistical power of Egger’s test is inherently limited when the number of included studies is small, as in our analysis (n = 10). With fewer studies, the test may fail to detect existing publication bias (Type II error). Therefore, the visual asymmetry of the funnel plot might be a more sensitive indicator of potential bias in this context. The funnel plot asymmetry could arise from several factors beyond just publication bias. For instance, it might reflect heterogeneity in study design, patient populations (e.g., variations in stroke severity or etiology), or assay methods used to measure ADAMTS13 levels. Smaller studies with null or less striking results might remain unpublished (the “file-drawer problem”), while larger studies or those with significant findings are more likely to be published, leading to the observed asymmetry. In conclusion, while the Egger’s test was not statistically significant, the funnel plot asymmetry cautions against definitively ruling out publication bias or other small-study effects. The overall interpretation of the meta-analysis on this association should therefore be made with this potential limitation in mind. Consequently, the extent to which our findings reflect the direct effect of ADAMTS13 versus the influence of these underlying conditions remains unclear. Future research should control for confounding factors.

## Conclusion

ADAMTS13 activity and plasma antigen levels were lower in patients with ischemic stroke. There was a negative association between ADAMTS13 levels and ischemic stroke, indicating that ADAMTS13 may play a role in the development of ischemic stroke. Likewise, patients who achieved unfavorable outcomes undergoing recanalization therapy had lower baseline ADAMTS13 antigen level rather than lower ADAMTS13 activity. Lower ADAMTS13 levels were associated with unfavorable early neurological outcome rather than 90-day prognosis. The conclusions drawn from this review are necessarily constrained by the limited literature available and the small sample sizes of several individual studies. Therefore, these findings should be considered indicative rather than definitive, and warrant validation in larger, future studies. Clinical trials evaluating recombinant human ADAMTS13 for cerebral ischemia treatment are essential. If confirmed in future prospective In review studies, a panel of blood biomarkers including ADAMTS13 might be a useful tool to guide recanalization therapies.

## Data Availability

The raw data supporting the conclusions of this article will be made available by the authors, without undue reservation.
